# Epistemic work and emotion in interprofessional practice: Lessons for interprofessional education

**DOI:** 10.1007/s10459-025-10438-3

**Published:** 2025-05-26

**Authors:** Rebecca E. Olson, Alberto Bellocchi, Louise Cooney, Diana Jones, Mark B. Pinkham, Bena Brown, Elizabeth Brown

**Affiliations:** 1 School of Social Science, University of Queensland, St Lucia, QLD 4072 Australia; 2 School of Teacher Education and Leadership, Queensland University of Technology, Kelvin Grove, Brisbane, QLD 4059 Australia; 3 Department of Radiation Oncology, Princess Alexandra Hospital, Woolloongabba, QLD 4102 Australia; 4 School of Medicine, University of Queensland, St Lucia, QLD 4072 Australia; 5 Southern Queensland Centre for Excellence in Aboriginal and Torres Strait Islander Primary Healthcare, Inala, QLD 4077 Australia

**Keywords:** Interprofessional practice, Interprofessional education, Video-reflexive ethnography, Epistemic cognition, Emotional climates, Radiation oncology

## Abstract

Use of theory to conceptualise interprofessional practice and inform interprofessional education is growing. This paper draws on two emerging theories in education and the sociology of emotions – epistemic cognition and emotional climates – to analyse an important interprofessional setting: weekly case conferences in one radiation oncology department. Drawing on detailed transcription of video data, ethnographic fieldnotes, and reflexive interviews with four participant/co-analysts, we analysed the knowledge aims, ideals, and processes for evaluating knowledge claims across 9 case conferences (3 meetings x 3 groups), as well as their associated emotional climates. Findings indicate that recency, and relational or disciplinary expertise are key values against which knowledge claims are judged. Epistemic styles and emotional climates vary; when meeting leaders encourage others to ask questions and promote a relaxed emotional climate, this may invite more diversified epistemic contributions. More broadly, our study brings together epistemic cognition and emotional climate as situated phenomena, providing empirical, conceptual and potential pedagogical advances.

## Introduction

Growing adoption of interprofessional practice (IPP) responds to the increasing complexity of patients’ healthcare needs, related to ageing populations and rising co-morbidities (Tsakizidis et al., 2016). It can also be seen to respond to several of the ‘quintuple aims of healthcare’: (1) reducing healthcare spending, (2) prioritising equitable access, (3) improving population health outcomes, (4) and patient satisfaction, and (5) protecting healthcare professionals’ wellbeing (Nundy et al., 2022). IPP is defined as “multiple health workers from different professional backgrounds provid[ing] comprehensive services by working with patients, their families, carers and communities to deliver the highest quality of care” (WHO, 2010). In short, IPP acknowledges that no single healthcare profession or professional has a full understanding of a patient’s and family’s embedded physical, sociocultural and emotional needs; thus, collective knowledge-sharing and decision-making is preferred. The rise of IPP has meant changes to the way health professionals are educated. Interprofessional education (IPE) refers to the training of pre-licensure health clinicians: readying them for IPP by learning “with, from and about” other health professionals (WHO, 2010).

A large body of scholarship has examined the effectiveness of IPE interventions in preparing students for IPP and in improving patient care (Reeves et al., 2013). However, much of this early IPE scholarship has been critiqued for two reasons. First, it draws heavily from cognitivist approaches to learning (Baker et al., 2021). Such approaches conceptualise IPE individualistically as knowledge, skills, or competencies (Sy et al., 2024). It is important that graduates have discipline-specific knowledge and knowledge of other professionals’ roles, and skills in teamwork. However, limiting the scope to cognitivist conceptualisations neglects the social and embodied nature of learning and IPP (Byerly et al., 2021). Second, it promotes a narrow view of IPP and patient outcomes as caused by IPE alone, neglecting contextual factors (Krystallidou et al., 2024). Neubauer and colleagues (2024), in particular, have been critical of such monocausal thinking, offering the term interprofessional organisations to recognise the importance of context-specific conditions to enabling IPP.

Many have argued for IPE scholars to attend better to the social complexity, such as power differences (Olson & Brosnan, 2017; Paradis & Whitehead, 2015; Stalmeijer & Varpio, 2023; Violato, 2022), and material contexts of learning and practice (Sy et al., 2024). To this end, there have been calls to employ theory more explicitly and robustly in health professional education, and IPE and IPP more specifically (Baker et al., 2021; O’Brien & Battista, 2020). Responding to this call, sociomaterial theories – such as situated learning theory and activity theory – have been used in recent years (Sy et al., 2024). Situated learning theory (Lave & Wenger, 1991) has been used in IPE studies to position learning holistically as a social process that occurs through practice and in context (O’Brien & Battista, 2020). Activity theory (Engström, 2001) has been used to articulate IPE learning activity systems, including subjects, objects, tools, communities, task divisions and outcomes (Byerly et al., 2021). And ecological theory has been used to recognise the messiness of IPP, with individual practitioners/professions always situated within affordances: ‘reciprocal relationships between resources in the world and the organism’ (Smith, 2023). Across these theories, there is agreement that IPP involves a coming together of knowledges, feeling bodies, material objects and environments, and (inter)actions for situated decision-making in healthcare.

Complementary to existing sociomaterial and ecological theory inroads into IPE and IPP, this paper responds to calls for ‘more and better use of theory in health professional education’ – including theories from disciplines outside of health professional education (O’Brien & Battista, 2020, p. 504). In this study we foreground two important elements of interprofessional decision making in one setting: knowledge and emotion. We capitalise on the growing interest into epistemic cognition within philosophy, psychology, and education research, by examining what the construct of epistemic cognition might offer IPE scholars. We complement our focus on epistemic practices with the sociological concept of emotional climates, to inform IPE and advance theorisation of IPP as emotionally imbued epistemic work.

## Background

To contextualise our study, in the paragraphs that follow we introduce the theoretical constructs of epistemic cognition and emotional climates.

### Epistemic cognition

Epistemic cognition refers to the process involved in thinking, justifying, and validating knowledge as true. Chinn et al. (2014) define it as ‘the complex of cognitions that are related to the achievement of epistemic ends’ such as advancing knowledge, understanding, and explanation. In short, epistemic cognition encompasses the cognitive work that is done to evaluate information as (in)accurate. Chinn et al. (2014, p. 52) helpfully deconstruct epistemic practices into three components, offering the AIR model (Table 1). However, Chinn et al. note that these components are interrelated in epistemic practices. Moreover, they emphasise the importance of epistemic aims being formulated before ideals and reliable processes become evident in a person or group’s epistemic activity.

**Table 1:** Chinn et al.’s (2014) AIR model of epistemic cognition

Component	Questions	Example
(A) Aims and values	What is the goal of the epistemic activity? What value is attached to this aim?	Wanting to find new or correct knowledge of a topic through • Truth-seeking • Understanding • Persuasion
(I) Ideals	What are the criteria for • Evaluating whether epistemic aims have been achieved? • Appraising the quality of epistemic products?	Evaluating the truth (A) of delivered testimony based on: • Coherence • Use of notes rather than recall alone
(R) Reliable (epistemic) processes	What methods are deemed to have a good likelihood of resulting in successful epistemic outcomes or producing epistemic products?	Cross examination, jury deliberation, judicial arbitration

Much of the scholarship on epistemic cognition foregrounds individuals and reasoning, drawing on quantitative or self-reported methods (cf. Muis et al., 2015). A review of scholarship examining epistemic cognition in medical education, for example, focused on how individual learners ‘activate personal theories of knowledge … which influence how they … make decisions,’ but called for research in naturalistic healthcare (rather than experimental or learning) settings (Eastwood et al., 2017, p. 1). Our application, in contrast, positions epistemic practices as inherently social and emotional. In clinical practice, information may be regarded or referred to as objects or artefacts. Our study foregrounds the relationality and social deliberation involved in IPP as an epistemic practice.^1^ This approach aligns with Kelly (2016, p. 394) who argues that what counts as knowledge is always situated within social practices. If we accept reliable epistemic processes as bearing a social origin, then emotions, which are individual and social phenomena (Denzin, 1984), are likely to have an important role in reliable epistemic practices. Thus, we also assess how the cognitive and social practices involved in epistemic processes interplay with emotions which help to ‘narrow[] down … the number of actions and consequences’ (Brun & Kuenzle, 2016, p. 458).

### Emotional climates

We supplemented theories on epistemic practices with the construct of emotional climates to overcome the limited attention to social and emotional dynamics within scholarship on epistemic cognition. Emotional climate emerges from longstanding theories on collective emotions that recognise how emotions spread in crowds through the synchronisation of vocalisations and physicality (cf. Le Bon, 1895). Often employed in studying the emotional dynamics of classrooms, the concept of *emotional climates* supports an understanding of emotions as collective and socially layered, with the affective tone of the institution or larger collectives shaping emotions and mood at the group level. The leader of the group (e.g., a teacher) plays an important role in shaping the group’s emotional climate (Bellocchi et al., 2014; Brackett et al., 2011; de Rivera, 1992; McKenzie et al., 2022). In this research, we define emotional climate as an overall emotional tone achieved by a group of interactants during situated encounters.

In analysing differing emotional climates, we draw on a concept central to the sociology of emotions: *collective effervescence*. Collins (2004) conceptualises collective effervescence as involving four ingredients necessary to achieving group solidarity: copresence, barriers to outsiders, shared mood and mutual attention. Like adding bicarbonate soda to water, ‘effervescence’ foregrounds the difficulty of containing emotion to one individual. Emotions are gassy and bubbly, spilling out of the individual and affecting others. Importantly, effervescent emotions do not need to be pleasurable; they need to be meaningful to a group’s shared mood, experience and identity.

In this study, we analyse the situated emotional and epistemic cognition practices enacted by groups of health professionals in one radiation oncology department based in Queensland, Australia. More specifically, we analyse *case conferences*: weekly meetings involving a range of health professions – radiation oncologists, occupational therapists, nurses, social workers, physiotherapists, speech pathologists, dietitians, and medical registrars – where clinicians review patients under their care to arrive at care plans. Case conferences are designed to address the complexities of caring for patients and their families, and are a key focus of IPE (Olson et al., 2022). Implicitly, they also have the potential to attend to the last of the quintuple aims of healthcare (Nundy et al., 2022), supporting healthcare professionals’ wellbeing: an aim that has grown in prominence following widespread COVID-related burnout, and that may be particularly important in cancer care settings. We analysed three different IPP teams’ case conferences to understand the groups’ emotional climates and epistemic practices and answer two research questions:

- What epistemic practices (aims, ideals, and reliable processes) for evaluating knowledge claims are employed across the three teams?
- How do epistemic practices and emotional climates interplay and vary across the three teams?

## Methods

**Fig. 1:**
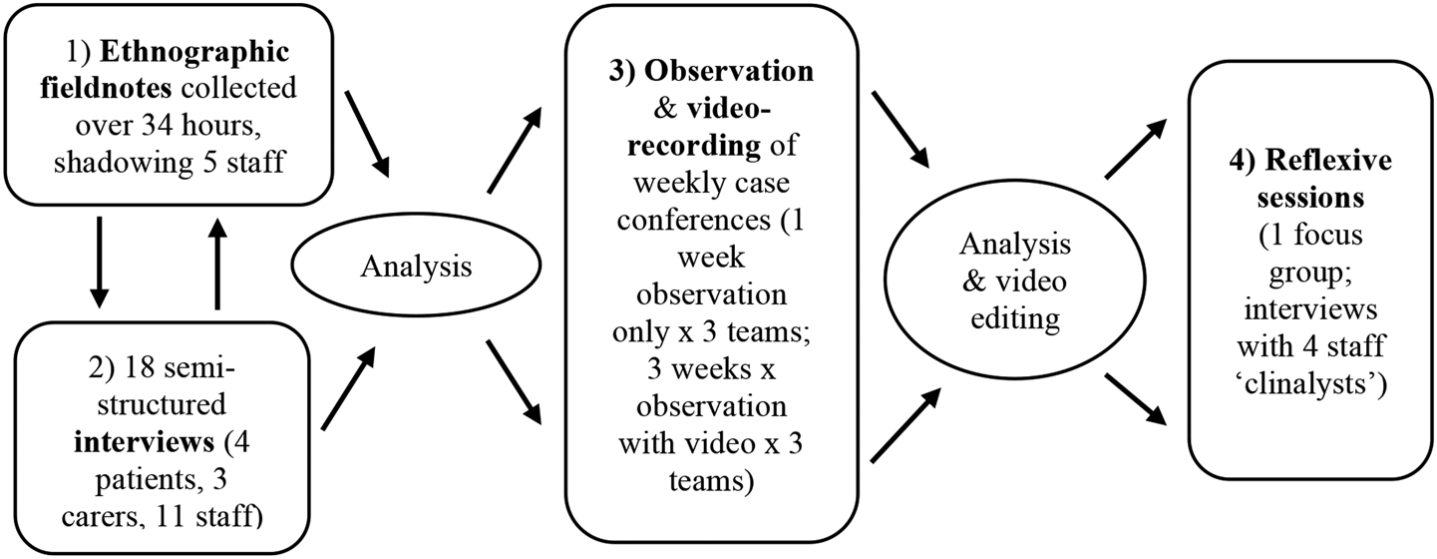
Study design

To answer our research questions, we drew on data collected as part of a broader study into emotions and IPP in cancer care (Olson et al., 2020). The study design was informed by video-reflexive ethnography: a qualitative methodology that invites those featured in videoed observations to join in the analysis to support participant-driven changes in practice (Iedema et al., 2019; Olson & Dadich, 2022). Reflecting the interpretivist and participatory aims of video-reflexive ethnography, and the value of their contributions – the clinician participants who contributed to reflexive analysis – what Carroll and Mesman (2018) call insider ‘clinalysts’ – are also co-authors. Methods of data collection supporting the broader project are depicted visually in Fig. 1 and reported elsewhere (Olson et al., 2020). The study received ethical approval through the relevant hospital ethics committee (HREC/16/QPAH/208) and university ethics committee (2016000666).

In this paper, we draw primarily on phases 3 and 4 (see Fig. 1). Phase three involved videoing weekly IPP case conferences led by three different senior consultants (3 videos x 3 teams = 9 video recordings). Table 2 provides an overview of the video-recorded case conferences. Note that twelve case conferences were observed (4 × 3 teams), but we delayed video-recording of the first case conference with each participating team, allowing participants to first become familiar with the researcher’s (Author 1) presence. All case conferences took place in the same meeting room within the hospital department. They were typically – but not always – led by the radiation oncologist. Using four GoPro^tm^ cameras positioned outward from the middle of the square meeting room table allowed us to capture the case conference’s social (spoken words, body language) and material (printouts, clipboards, pens and paper, and laptops) elements.

**Table 2:** Videoed case conferences

Senior consultant	Observation identifier	Team composition	*n*	Length of recording (mins)
‘Sam Tulloch’ (ST): leader of Team 1	1.1	RadOnc (lead), Reg, Nurse, SW, PT, Jr OT	*n* = 6	33:53
	1.2	Reg (lead), Nurse, Diet	*n* = 3	18:49
	1.3	RadOnc (lead), Nurse, SW, Reg, OT, Jr OT	*n* = 6	24:46
‘Oli Rakos’ (OR): leader of Team 2	2.4	RadOnc (lead), Nurse, OT, PT, SW, SP, Jr Diet	*n* = 7	21:11
	2.5	RadOnc (lead), Nurse, SW, OT	*n* = 4	15:31
	2.6	RadOnc (lead), Nurse, SW, PT, OT, Nurse Const, Diet, SP	*n* = 8	24:02
‘Kerry Sussman’ (KS): leader of Team 3	3.7	RadOnc (lead), Nurse 1, Nurse 2, Reg, SW, PT, OT, Jr Nurse	*n* = 8	38:24
	3.8	RadOnc (lead), Nurse, OT, PT, SW, Reg	*n* = 6	25:13
	3.9	Reg (lead), Nurse, SW, PT, OT	*n* = 5	29:18

Key: Diet = Dietitian; Jr Diet = Junior Dietitian; Jr Nurse = Junior Nurse; Jr OT = Junior Occupational Therapist; Nurse Const = Nurse Consultant; OT = Occupational Therapist; PT = Physiotherapist; RadOnc = Radiation Oncologist; Reg = Registrar; SP = Speech Pathologist; SW = Social Worker

Phase four was useful to checking and extending our analysis. This phase involved: (1) one reflexive focus group to discuss video excerpts and interview quotes with staff featured in data collection, and (2) reflexive interviews with four staff participant ‘clinalysts’ to extend reflexivity and analysis (1 senior consultant and 1 nurse interviewed together; 2 allied health professionals interviewed independently).

To ready our video data for analysis, we first used Adobe Rush^tm^ or V-Note^tm^ to synchronise and review the videos from each case conference. At this point, Authors 1 and 2 selected emotional climate and epistemic cognition as useful theoretical avenues, prompting us to refine our research questions. Author 1 then transcribed the video data, following ethnomethodological traditions: recording the words, movements, and facial expressions of all case conference meeting contributors (Garfinkel, 1967). Following transcription standards, non-verbal movements including facial expressions are indicated within double brackets ‘(( ))’, pauses are indicated using single brackets ‘(# sec pause)’, omitted transcriptions are indicated using an ellipsis ‘ …’ and overlapping text is indicated with ‘#’ (Flick, 2023). For brevity, non-communicative utterances (e.g., ‘um’s) have been omitted. Any included names (of senior consultants) are pseudonyms; potentially identifiable content has not been included.

Having prepared accurate transcripts, our analysis of epistemic cognition and emotional climate unfolded separately. In our first pass, authors 1 and 2 independently coded the aims (*A)*, ideals (*I*) and reliable processes (*R*), following Chinn et al.’s (2014) model. We often worked backgrounds within transcripts from the epistemic product (e.g., a care plan), to identify what processes the IPP teams relied upon to create those products. Using clean transcripts, in the second pass, we identified the emotional climate within each team meeting. This was initially achieved through subjective interpretation. Subsequently, we drew on two methods that offered objective means to rate group climates: turn-taking; and power in the air.

Following Nugus et al. (2010), we took account of each turn taken within case conferences. Turn taking was calculated using a table to count how many turns were taken by each case conference attendee, and the purpose of each turn. Analysis of turn-taking yields understandings of the distribution of turns at talk and therefore power-structures of IPP meetings, which are indicative of the kinds of emotional climates within groups. Power in the air refers to the intensity (amount of energy passing a point at a given time) generated through vocalisations (Boersma, 2002; Roth & Tobin, 2010). Power in the air served as a proxy for emotional climate based on measures of sound energy produced by the groups (cf. Roth & Tobin, 2010). Calculating power in the air allowed us to compare within-team emotional climate and between-team emotional climates.

Once epistemic cognition and emotional climate were coded separately, we then brought our coding together to identify how epistemic cognition unfolded in teams that displayed different emotional climates. Finally, we compared our interpretations of epistemic cognition and emotional climate to those discussed by staff during interviews, drawing on transcriptions from reflexive interviews to confirm or extend our understanding of the work and value of emotion within case conferences.

In using naturally occurring data, we accept that our analysis infers from our observations and interpretations of situated actions. We also accept that author 1’s presence during case conferences may have affected how team members behaved. Her prolonged engagement in this setting partly reduces this effect (accounts from reflexive interviews conducted as part of this study offer confirmation). However, we also acknowledge that there is no other way of coming to know the IPP teams’ epistemic processes as enacted practices if researcher presence and inference is not a part of the methodology. Moreover, we also draw on interview data to understand the IPP participants’ perspectives on salient aspects of their interactions, using both first and third person perspectives.

For clarity, we separate our results into two parts: epistemic practices, followed by emotional climates and how they intersect with epistemic practices.

## Results part 1: Epistemic practices

In the first part of our results, we draw on Chinn et al. (2014) to identify the epistemic aims (A) related to ‘cases’ discussed in the case conferences. We then present epistemic ideals (I) and reliable processes (R) together, as we found that reliable processes *evidence* the ideals used when evaluating information or knowledge. Some of the aims presented here may appear obvious to experienced clinicians. However, establishing these aims – common and uncommon – is useful to IPE, necessary to applying Chinn et al.’s model, and helpful to developing our argument that IPP involves *emotional epistemic climates* (E-E climates).

### Epistemic aims of case conference meetings

In case conferences, typical epistemic aims involved discussing a patient’s diagnosis, treatment, and circumstances to gain a better understanding of the patient’s condition and make care management-related decisions. Thus, epistemic aims involved time-sensitive data sharing, questioning, or better understanding data relevant to the patient. Temporality was a key reason for these meetings: to ascertain if available notes are out of date, to assess which is the most up-to-date information and share it across the team, to clarify which patients are coming into their care, current or shifting to other departments. There was also a focus on the patient’s temporality. The time the patient has left – in the care of the radiation oncology department and on this earth – was a priority in the meetings, underpinning what information was needed when. Furthermore, chronological time was a key measure of variation across patients discussed in case conferences, with some covered in under half a minute, and others the focus of cross-team discussion for several minutes.

Foregrounding epistemic practice and temporality, here we analyse the epistemic aims – and, where relevant, the connected ideals, reliable processes and consequences – of weekly interprofessional case conference meetings within one radiation oncology department. Overall, in our analysis, we identified two key epistemic aims that were achieved by the IPP groups: (1) rapid information sharing, and (2) questioning or extending understanding of information. Below we show how these aims and associated reliable processes and ideals were enacted through the situated practices of IPP teams.

#### Epistemic Aim 1 - Rapid information sharing

Cases with an epistemic aim of information sharing were often very brief. Here, patient data was treated as an object for presentation, rather than artefacts for scrutiny in the co-production of a care plan. The goals in such exchanges were limited, often because the consequences within the team’s control were limited. These brief exchanges typically fell into two categories: (1) patients outside of or not yet in the care of the department; and (2) patients with few care needs. From case conference 3.8, led by radiation oncologist KS, we see an example of the former. In the span of 48 s, KS verbalises the name, ward location, diagnosis, symptoms and type of radiation for three patients who were either outside of the team’s oversight or not yet within their care. The key aim here seems to be raising clinicians’ awareness and giving them a frame of reference for integrating future information on the patient.

In case conference 1.3, led by radiation oncologist ST, we see an example – lasting less than thirty seconds – of rapid sharing information on a patient with few care needs.

**Table:** 

RadOnc	[Patient name], [diagnosis]. So far so good, so they are in the second week of six. They are going fine, they have had some mild symptoms which are nothing of major concern so far. They had some eye symptoms which is one of the potential side effects associated with the experimental drug ((gesticulates as if pointing to different phrases within a sentence)) that is part of the trial they’re on
SW & Nurse	((make notes))
RadOnc	so we’ll keep an eye on that but no big deal at this stage, just monitor. It is worth everyone catching up with them but there are no pressing issues

Here we identify the epistemic aim as informing the team of the relatively new patient’s ‘mild …. eye symptoms’. The knowledge claim that the new symptoms are a side-effect of an experimental drug is not judged against any ideals. Perhaps this is because that is not the expertise of the team or because few team members had met with the patient at the time of the meeting. The epistemic aim unfolds quickly into a broad and non-urgent consequence: a direction for allied health clinicians to ‘monitor’ and meet with the patient in due course.

Thus, we found the epistemic aim (A) of rapid knowledge sharing led to the epistemic consequence of future knowledge-seeking and monitoring. This meant no deliberation or evaluation against ideals (I) of the knowledge-shared was required in the present.

#### Epistemic Aim 2 - Questioning or extending understanding

Cases where epistemic aims related to better understanding data relevant to the patient’s treatment and circumstances required longer deliberation. In the next excerpt (case conference 3.8), radiation oncologist KS introduces a patient with cancer who also has a separate chronic health condition. The initial aim appears to be information sharing. As the interaction progresses, questioning unfolds. The OT seeks to clarify if there is an epistemic consequence and how to achieve it: visiting the patient on the ward or within radiation oncology.

**Table:** 

RadOnc	[Patient name] is a person with [medical condition]. They have been [de-identified] nearly all their life. They’ve been found to have [organ] cancer. It’s a particularly nasty one …. They tried initially to give some chemotherapy which they tolerated with recurring infections. The patient is not a surgical candidate, so they’ve come to us for palliative radiation ((nods, sympathetic smile)). They have a partner and they are from a nursing home …. So, they are very sort of institutionalised
OT	So, we need to see [patient name] on the ward?

In this excerpt, KS’s initial epistemic aim is to share the patient’s circumstances. Following the OT’s request for clarification, the epistemic aim shifts to understanding the patient’s location. While the printout indicates the patient is on the ward, a recent conversation with staff from the ward gives the radiation oncologist cause to question the printout’s veracity.

**Table:** 

RadOnc	The patient is on the ward ((points to printout)) yeah but I’m not sure, they were planning on discharging them ((furrows brow, waves hand with open palm)) in the very near future
OT	They had a weakness fall on [date]
RadOnc	Which was ((looks at watch)) #yesterday#
OT	#Yesterday#
RadOnc	Oh gosh ((rests head in hands, elbows on table))
OT	… ((reads from laptop)) The patient was found lying supine on the floor, hands to head …. That sounds like. The patient went to see psychiatry maybe while they were in there? …
SW	Who’s the patient in there with?
OT	Its [department name]. ((reading)) Febrile on [ward name]. Continue antibiotics. No other concerns. So, there’s not really an indication of discharge as yet, but
RadOnc	Right

Here the team rely (R) on the case conference process to compare and appraise (I) epistemic artefacts (i.e., the printout), addressing the epistemic aim (A) of deciphering if the patient has been discharged. The process they rely on involves a comparison of (1) the information on the printout, with (2) the radiation oncologist’s recall, and (3) the information entered into the patient’s electronic medical record. Following Chinn et al. (2014), this exchange can be inferred as drawing on the epistemic ideals of using empirical evidence, looking for coherence across explanations, and applying standards of (expert) testimony. This ideal along with the enacted search for converging information represent the team’s reliable processes for generating accurate knowledge about the patient’s discharge status.

Next, with the aim of deciphering the patient’s location resolved, the epistemic aim shifts to clarifying the epistemic consequence: who within the team needs to see the patient.

**Table:** 

OT	It doesn’t look like any of us have been involved yet. [name of other OT]- oh there’s physio review
RadOnc	((looks to PT))
PT	Of course ((smiles))
SW	((smiles, highlights on printout))
OT	((laughs)) And pharmacy, that’s it
RadOnc	((flips over page on printout)) Okey dokey. Okay. We’d better head up

Here, the OT reviews the patient’s electronic medical record to conclude that everyone in the team but the physiotherapist needs to meet with the patient. The social worker highlights on the printout, perhaps as a reminder to follow through on the epistemic consequence. Overall, there is a high reliance on epistemic artefacts: the printout and records accessed through the OT’s laptop.

This finding suggests that IPP within this radiation oncology department involves time-sensitive decision-making. Data is always timebound, justifying the regularity of case conferences. Judging the accuracy of information is also reliant on the individual disciplinary expertise of each member who generates information (via reports stored on databases) about the patient’s situation. As a patient’s health changes over time, data requires updating. For this reason, the information from databases require scrutiny. In this case, a team member who has most recently seen the patient, has most recently spoken with one of the other relevant specialists about the patient, or – as illustrated in the next excerpt – has the best rapport with the patient, may have the most reliable information.

In the following excerpt (case conference 2.6 led by OR), the epistemic aim is to question the patient’s understanding of their treatment and thus their informed consent. Here, we see other epistemic ideals employed, through enactment of reliable processes which these teams use in making epistemic judgements. Namely, the processes involved a valuation of testimony from clinicians with a good understanding of the patient, due to their professional expertise, recent interaction, and/or relationship with the patient.

**Table:** 

RadOnc	Now [patient name]. Now [SW]
SW	((looks up to make eye contact with RadOnc))
RadOnc	I want to speak to you about [patient name] …
	((takes glasses off)) the patient is a lovely person but just doesn’t ((folds glasses with left hand and chin)) seem to- We had issues at the beginning with their consent. The patient didn’t understand what was going on. So we effectively had to re-consent them
OT	((searching on laptop))
RadOnc	… I’m just not sure whether the patient really has any full comprehension of what’s going on. So, I wouldn’t mind if you could chat, catch up with them at some stage
SW	((nodding))

Within the first thirty seconds of this 2-minute-and-24-second-long interaction, the RadOnc’s epistemic aim is clear: to check if the patient understands the prescribed treatment and is therefore able to provide consent. We can infer that there is a high value placed on this aim, evidenced by the relief that the radiation oncologist describes in the next excerpt.

**Table:** 

SW	Well, I did speak to the patient
RadOnc	Oh good
SW	this morning
RadOnc	Oh good ((places glasses on table))
SW	And
RadOnc	Yes
SW	the patient was really happy with the way you explained everything
Nurse	Aww ((looking at RadOnc, smiling with open mouth))
RadOnc	((smiles)) Oh okay. Thank you. Well, you put my mind at rest ((leans back in chair and puts both hands on heart, then returns to lean both arms on table, laughs))

Here we see rapid resolution of the epistemic aim. The social worker has confirmed that the patient understands. The assessment is not questioned by the radiation oncologist or any other team member. The prompt acceptance of the social worker’s testimonial reflects an ideal. The social worker’s epistemic authority is judged to be valid, perhaps due to their professional training, as well as their relational and opportunistic knowledge. This interpretation was supported within our reflexive interviews, where staff underscored the value of ‘personal knowledge of engagement with that person.’ Continuing with the excerpt from observation 2.6, the occupational therapist then joins in the interaction, supporting the social worker’s assessment.

**Table:** 

RadOnc	They’re [the patient] a bit. I think they’re quite simple
OT	Yes
SW	Yeah. It
RadOnc	You met the patient?
OT	We know the patient from the [department name] Service
RadOnc	Yeah
OT	And I actually just saw the patient
RadOnc	Yeah
OT	On Monday and they said exactly the same thing
Nurse	Aw, [RadOnc] ((smiles and looks from RadOnc, back to OT, clicks pen))
OT	The patient felt that things were a lot clearer …. The patient is special in that they need a little bit more time to understand things ((smiles))
SW	Yes ((nodding))
RadOnc	Okay
OT	in their own way, but I think the patient does. They were able to fully explain everything [treatment/care] that was coming up. ((raises eyebrows, nodding)) ….
RadOnc	…. Good because it’s been worrying me a bit whether the patient knows what’s going on. ((briefly leans back in chair and then returns to lean on table)) ….

In this excerpt, OR questions the basis of the occupational therapist’s assessment: ‘You met the patient?’. Through this questioning, the epistemic ideals can be inferred. The basis on which the occupational therapist’s claim is judged is their relational and opportunistic knowledge of the patient’s understanding. Following resolution of the epistemic aims, the consequences – the action resulting from the purposeful deliberation of knowledge claims – can be shared. In the next excerpt, the epistemic consequence is declared: a plan to move forward with treatment, now that it is confirmed that it is ethical to do so. However, the plan is not a stable one, it is contingent on successful equipment repairs.

**Table:** 

RadOnc	…. So the plan with this patient is we… are going to treat their [body part] because we can do that now. And the patient has got two [body location] secondaries from [organ] cancer as well which are very suitable for stereotactic treatment. But, we can’t do that at the moment because the machine that treats the stereotactic is having a (1 s) #uh- a refit#
SW	#Oh. Yes. That’s right# ((opens mouth, closes mouth, makes note on printout))
RadOnc	[Code number] So when that refit is done, we will do the patient’s [body location]

Therefore, in cases where the epistemic aim (A) relates to questioning or extending understanding, there is reliance (R) on the interprofessional case conference process, use of props or artefacts that are brought to the meetings (e.g., laptops, printouts), and input from the clinicians present. Through this process, evidence and testimony are scrutinised against ideals (I) of coherence across explanations and other evidence. Other epistemic ideals include:

1. The expertise of individual professionals, based on their relationship with the patient, experience and/or disciplinary knowledge as well as their documented evidence;
2. Situated knowledge from any professional about the latest condition of the patient.

Resolution of scrutiny then leads to an epistemic consequence: commonly, a temporary patient care plan.

These points represent the nature of the reliable epistemic processes witnessed during case conferences. In point 1, a professional’s disciplinary expertise or in-depth knowledge based in extended interactions, and capacity to apply this in the assessment of a patient’s condition is that individual’s *authority.* The interprofessional team is highly reliant on this authority. Reliance on databases during case conferences reveals that *trust* in expert authority (i.e., disciplinary or relational knowledge + patient assessment) forms one of the fundamental epistemic practices and values of the group. However, due to point 2, the *temporality of information*, any static report such as a database entry may not be sufficiently up to date; the epistemic practice of challenging information in databases based on more recent patient contact, trumps reliance on and trust in static reports.

## Results part 2– emotional epistemic climates

### Interpreting emotional climates

Aligned to the epistemic practices above, we also observed differences in emotional tone across the interprofessional teams. The data display below evidences the emotional tone of KS’s case conferences, with the team expressing a collective ‘aw’.

**Table:** 

RadOnc	[Patient name] is an elderly person with a [organ] cancer … They’ve had one cycle of chemo. They are now on radiation. I met the patient with their children. The patient has now moved into the nursing home part of the nursing home, close to their spouse
OT	Great ((nod, sighs))
RadOnc	So, the patient’s children are very happy about that. ((touches right hand over left hand))
Nurse	and apparently despite the spouse’s [medical condition], they haven’t seen each other for many weeks
RadOnc	Ohhhh ((looking at Nurse, furrowed brow, smiling))
Nurse	and the spouse was so excited when they saw the patient
All	((wistful “aw”s and sighs around the table))
OT	That’s lovely
RadOnc	…it’s like, has everyone seen that film *The Notebook* ((brings hands up to forehead, covers face briefly, then laughs))
All	((agreement noises, laughter and understanding))
OT	Stop I’m going to cry! ((emphasises, points with hand))

The empathy culminating in near tears in the above extract sharply contrasts with case conferences run by OR, where teasing and laughter were commonplace. This is illustrated in the data display presented above from case conference 2.6 where the social worker and nurse are observed teasing OR. The social worker belabours the point that the patient now understands the proposed treatment plan due to the radiation oncologist taking time to explain it to them, saying ‘The patient does [understand] now … Thank you [OR]’ before leaning back in their chair and smiling. The nurse also teases, saying ‘Aw, [OR]’ and smiling; and later on, says, ‘have a chocolate’ as a reward for the clear explanation. This teasing from the social worker and nurse provoke smiles from the team. Case conference 2.6 was not the only observation where we witnessed teasing within this team.

During case conference 2.4, led by OR, data displays show the radiation oncologist teasing the allied health clinicians. In this first excerpt from 2.4, the team discusses the second patient on their list, who requires a radiologically inserted gastrostomy (RIG): a tube inserted into the stomach of someone experiencing swallowing difficulties, allowing fluid intake. OR teases the speech pathologist that the treatment outcome all depends on them.

**Table:** 

RadOnc	So [patient name] ((looking down at printout, touching printout with right hand)) is the person with the [organ] tumour who has a RIG and who I think we’re going to struggle with but [name of SP] is going to get them through treatment
Nurse, SW	((look up at SP, smile, quick laugh))
SW	Go [name of SP]! ((shakes fist in enthusiasm, smiles at SP, laughs))
OT	Nice work SP
SP	It’s just all up to me ((laughs, leans back in chair))
RadOnc	Hey?
Nurse	Yes, Nobody else ((smiles, laughs, returns to making note on printout))

Such joking was described as important within reflexive interviews: ‘in order to work in this space, it’s [laughter] really a powerful tool to keep us moving forward and connecting within that space’ (Dietitian). It served as a relief from more serious conversations, such as the team’s subsequent discussion of a patient at imminent risk of death, and OR’s sustained concern over how best to deliver a terminal prognosis. In this next excerpt from case conference 2.4, the radiation oncologist implicitly but repeatedly seeks affirmation from the team on how to deliver the prognosis.

**Table:** 

RadOnc	Next we have [patient name]. Now [patient name] is a person with [organ] cancer involving their [body location] … They have had treatment there before. They have a big hole there which looks very nasty …. some time I am going to have to tell them that that their [organ] cancer is going to erode into their [body location] artery
SP	((brief grimace, drawing corners of mouth down, looking at RadOnc))
RadOnc	and it is going to be a quick exit
OT	((moves print out, then stops to look directly at RadOnc when OR says ‘quick exit’, makes note on print out))
RadOnc	… Because I don’t think we are going to control this tumour and if it’s not controlled that’s what it’s going to do
Nurse	((raises eyebrows up in the middle, indicating sadness and sympathy, nods)) Yeah
RadOnc	But it’s probably better than you know dying a slow death with a huge [de-identified] ((rolls hands)) messy [body location] ….
PT	I saw them this morning. The patient is well set up at home. They have a stair lift. The patient’s child is quite involved. The patient was on the ward recently a few times. Has a stick and a wheelie walker. I think it’s just the dressings ((points pen towards Nurse)) that are #stressing the patient out a bit#
Nurse	#Dressing and# they had a bit of flare pain yesterday in [body part] and in their [body location] but we just- ((nods))
RadOnc	… But anyway, I will I’ll just have to have a hard chat with the patient and tell them …

Clear differences in the emotional tones within case conferences and across the interprofessional teams were observed in the data. Group leaders (typically the radiation oncologist, but sometimes – in their absence – the registrars) reflected different communication and interactional styles. These differing styles clearly informed the groups’ emotional climates. One reflexive interviewee described this as important, saying ‘radiation oncologist personality or tone can influence things significantly.’ Other likely influences include cancer type and treatment modality (curative, adjuvant, or palliative) of the patients discussed in the case conferences. Differences in cancer type were noted across the teams (radiation oncologists within the department are typically assigned patients with one or two cancer types), with some more often treating patients with complex supportive care needs (e.g., patients with young families). Broad treatment modalities, however, were observed across all teams. Furthermore, it is likely that group emotional climates were also shaped by team cohesion, trust, and length of shared experience.

Overall, our subjective experience of the differing climates can be summarised using the terms *jovial* for case conferences led by OR, *sombre* for those led by KS, and *subdued* for those led by ST. A jovial climate was represented by frequent use of humour, laughter and a louder soundscape. In contrast, sombre climates were characterised by leaders who spoke softly and slowly. Softly spoken humour sometimes features, but more often leaders evoked shared sadness and awe. Subdued climates included moments of laughter and shared sadness but were on-the-whole less emotional and more efficient. It is important to note these are general assessments of the emotional climate of a case conference session. More sombre case conferences still had jovial moments; likewise, more jovial case conferences had sombre moments.

The differences in the emotional tones across all teams goaded us towards further analysing these emotional climates. We attended to the collective emotion produced in each of the case conferences, and how this collective emotional arousal interacted with epistemic processes. Thus, we examined auditory intensity and correlated turn-taking.

### Soundscapes

Extending on our experience and interpretation of the different climates, we ran acoustic analyses of audio data from each case conference using PRAAT software (see Table 3). Sound files for each team (i.e., 1-ST, 2-OR, 3-KS) were analysed to extract values of sound intensity in decibels (dB). Table 3 presents the lowest and highest intensity values from the nine observations of the three case conference teams.

**Table 3:** Low and high ranges of sound intensity in IPP audio files

Observed meeting		Intensity range (dB)	
Identifier	Audio file code	Low	High
1.1	ST2303	62.66	64.8
1.2	ST3003 (Registrar)	59.94	63.66
1.3	ST2004	61	63.53
2.4	OR2505	66.5	72.34
2.5	OR0106	66.08	70.28
2.6	OR2206	67.36	72.25
3.7	KS0607	60.41	71.74
3.8	KS1307	60.9	64.55
3.9	KS2007 (Registrar)	59.85	64.28

As can be seen in Table 3, our subjective experience of different climates across the groups is supported by differences in high and low intensity values, with two of the OR group meetings – 2.4 and 2.6 – representing the two highest intensity values recorded, with only one group meeting (3.7), by a leader other than OR, reaching a value above 70 dB. In contrast, the two sessions led by a registrar,^2^ in which we experienced the most subdued climates, have the lowest recorded values, aligned with intensity levels (60dB) considered by linguists to be just within normal conversation levels (Koffi, 2020). We do not claim that our data represent absolute measures of the meeting soundscapes. However, these data provide transparency in reporting our perceptions of the different climates produced by the groups.

### Turn-taking

Finally, we connect the emotional (and auditory) climate of the case conferences to their interprofessional epistemic practices by analysing turn-taking. We will show how, overall, IPP teams where a greater number of members contributes to talk have less hierarchical epistemic structures, aligned with positive effervescent climates. Below in Tables 4, 5 and 6 we compare the turn-taking – and purpose of each turn – across three carefully selected case conferences. Meeting 1.3 was selected as a case conference led by a radiation oncologist (ST) with a more subdued emotional climate. Meeting 2.4, led by OR, was selected as a case conference with a jovial emotional climate. Meeting 3.7, led by KS, was selected as a case conference with a sombre emotional climate. It is important to note that within reflexive interviews, participants described time-pressures associated with the case conferences led by ST that likely shaped the emotional climate. Another senior consultant held case conferences directing after ST in the same room, so it was not possible to run over-time.

**Table 4:** Turn-taking in case conference 1.3 (Subdued)

	No. of times information offered	No. questions asked	No. questions answered	No. of directives given	No. of directives accepted	No. of jokes made	Cumulative No. turns taken	(Background talk)
RadOnc	35	13	8	10	0	1	**67**	0
Nurse	10	8	9	0	4	2	**33**	0
SW	5	0	1	0	1	1	**8**	0
OT	2	2	1	0	3	1	**9**	2
Jr OT	0	0		0	0	0	**0**	2
Reg	3	0	4	0	0	0	**7**	0

Comparing the tables, in all three case conferences radiation oncologists take the most turns, followed by nurses. This is unsurprising. With the way case conferences are set up, radiation oncologists introduce each patient on the list and therefore would be expected to take the most turns. Given the strong value placed on knowledge derived from interactions with patients, it is not surprising that nurses take many turns in delivering information and answering questions. Across each team, nurses typically are the health professionals who spend the most time – sometimes hours – with patients and their families/carers following radiation therapy.

**Table 5:** Turn-taking in case conference 2.4 (Jovial)

	No. of times information offered	No. questions asked	No. questions Answered	No. of directives given	No. of directives accepted	No. of jokes made	Cumulative No. turns taken	(Background talk)
RadOnc	26	7^1^	13	2	0	4	**52**	0
Nurse	8	5	9	2	1	0	**25**	0
SW	2	5	1	0	2	0	**10**	0
PT	1	2	6	0	0	1	**10**	0
OT	5	5	1	0	0	0	**11**	0
Jr Diet	0	0	0	0	0	0	**0**	1
SP	3	3	0	0	1	2	**9**	1

^1^ Note: Directive given as question 3 times

There is, however, a noticeable difference in direction giving, question asking and overall turn-taking across the three tables. In case conference 1.3, all ten directives are delivered by the radiation oncologist. In case conference 2.4, there are four directives given, and these are shared between the nurse and the radiation oncologist. An additional three directions are posed as questions by the radiation oncologist. In case conference 3.7, 6 directives are given, with one emerging from the radiation oncologist and the remaining five from the social worker. Asking questions (and epistemic aim setting) is also more equally distributed in Tables 5 and 6. In case conference 2.4, 7 out of 27 questions are posed by the radiation oncologist. In case conference 3.7, 4 out of 20 questions are posed by the radiation oncologist, compared to 13 out of 23 in case conference 1.3. Overall turn-taking is also more evenly distributed across case conferences 2.4 and 3.7. While the social worker – for example – in case conference 1.3 takes only 6% (8/124) of the turns, in case conferences 2.4 and 3.7 the social worker takes 8.5% (10/117) and 15% (22/147) of the turns. Furthermore, while junior health professionals do not openly contribute, beyond side conversations with the health professional next to them (categorised as background talk) in case conferences 1.3 and 2.4, the junior nurse in case conference 3.7 contributes twice.

**Table 6:** Turn-taking in case conference 3.7 (Sombre)

	No. of times information offered	No. questions asked	No. questions Answered	No. of directives given	No. of directives accepted	No. of jokes made	Cumulative No. turns taken	(Background talk)
RadOnc	43	4	8	2	1	2	**60**	0
Nurses (2)	25	5	9	3	0	0	**42**	0
SW	3	9	1	2	5	2	**22**	0
PT	8	0	2	1	0	0	**11**	0
OT	3	1	0	1	0	0	**5**	0
Jr Nurse	0	1	1	0	0	0	**2**	0
Reg	5	0	0	0	0	0	**5**	0

Overall, these findings suggest that IPP involves emotional epistemic climates. In groups where an effervescent climate – sombre or jovial – is observed, there is openness in interactions (i.e., more members taking turns-at-talk). In case conferences with more effervescent emotional climates, there appears to be more surfacing of epistemic aims and more scrutiny across the interprofessional team of the veracity of any information in the context of its temporal dimension. Claims to knowledge about a patient’s condition are tested by more diverse group members. This may be because clinicians feel more comfortable contributing in case conferences with more effervescent emotional climates, and flatter turn-taking structures. In such settings, the epistemic community (the interprofessional team) shares an emotional climate that extends their epistemic practices. One could even go as far as suggesting that the epistemic practices may be more reliable in interprofessional case conferences where the emotional climate allows for comfortable and non-confrontational challenges to and questioning of information.

## Discussion

Through this empirical study of IPP teams in one radiation oncology department, we advance a theory of IPP as involving *emotional epistemic climates* (E-E climates). In IPP case conferences, enhanced epistemic practices are produce by teams with effervescent climates characterised by greater turn-taking from more diverse team members. That is, the epistemic practices of creating, sharing, and evaluating knowledge in working towards a patient care plan within IPP are enhanced by more effervescent emotional climates. More effervescent climates are characterised by jovial and sombre moods and greater turn-taking by individual members. We theorise that the increased turn-taking results from greater comfort experienced by team members in more effervescent climates. In turn, this leads to more diverse group member participation, which can enhance patient care planning through recency of information and a wider range of processes being engaged in validating patient and care information. This theorization will require further conceptualisation and empirical testing in future research. Although our findings will be of interest to scholars of epistemic cognition, this discussion prioritises implications for the intersecting scholarship on IPP and IPE.

### Interprofessional education & practice

First, our study responds to calls to extend theorisation of IPP. Such calls urge engagement with disciplines beyond health professional education: to inform IPE (O’Brien & Battista, 2020) and foreground the ‘affective aspects’ of IPE and IPP (Sy et al., 2024). This study builds on the work of Gordon et al. (2017) to show the merits of drawing on naturally occurring video-based and video-reflexive data to extend theorising of IPP and IPE. Our theorisation – which draws on concepts used in sociology, philosophy, and educational psychology – offers an appreciation of IPP as involving socio-emotional and epistemic cognition practices that is complementary to socio-material theorising. Socio-material theorising of IPP highlights the relationality of humans and materials, to conceptualise IPP as a culmination of mental, embodied, and material interactions (Sy et al., 2024). Emotions are acknowledged in socio-material theories of IPE and IPP, but tend to be backgrounded.

As part of our extended theorising of IPE/IPP, our study elevates emotional climates alongside epistemic deliberations as two important elements worthy of sustained scrutiny given the importance of emotions to healthcare, IPP, and teaching and learning (Boler & Zembylas, 2003; Cottingham et al., 2018; Dadich & Olson, 2017; Sy et al., 2024). Findings suggest that particular emotional climates may be more conducive to power sharing, which has repeatedly been identified as a challenge within IPP and IPE scholarship (Baker et al., 2011; Nugus et al., 2010; Paradis & Whitehead, 2015; Stalmeijer & Varpio, 2023; Violato, 2022). Foregrounding emotions may be especially useful in the current context, where clinician burnout is widespread. Within reflexive interviews, staff described moments of connection and comedic relief as important to countering the gravity and uncertainty of cancer care, and increasing the sense of unity, connection and belonging within the team. Future research should examine how emotional climates in IPP might intersect with the fifth aim of healthcare – protecting healthcare professionals’ psychosocial wellbeing (Nundy et al., 2022) – and how IPE can better prepare clinicians in prioritising psychosocial wellbeing through attending to emotional climates.

Second, our findings support understandings of IPP – and by extension revisions to IPE – as practices situated within interprofessional organizations. Dominant cognitivist approaches within health professional education position IPE as an accumulation of knowledge and behavioural competencies (Baker et al., 2021; Krystallidou et al., 2024; Sy et al., 2024), reinforcing a ‘narrow definition of teams and teamwork’ (Thistlethwaite & Xyrichis, 2022, p. 165). In such approaches, developing competencies through IPE is thought to cause effective IPP (Neubauer et al., 2024); emotions are typically deemed as irrelevant, or worse pathologised (Ajjawi et al., 2022; McNaughton, 2013). Counter to dominant approaches, we support a broader, less individualistic and more socioemotional appreciation of IPE and IPP.

Adding to this, we support calls for IPE to focus on more than competency development. IPE should prioritise supporting learners to read interprofessional contexts and deploy their (embodied, cognitive and behavioural) IP skills in context to co-create more a/effective IPP. Such an approach complements scholarship which recognises the importance of interprofessional settings as barriers or enablers of IPP (Neubauer et al., 2024). It also supports calls for paradigmatic pluralism in IPE (Baker et al., 2021), recognition of the social aspects of learning, and application of adult learning practices such as reflection (Bogossian et al., 2023). Extending on Byerly and colleagues (2021), this IPE curriculum could prioritise reflection following work-integrated or simulated IPP on roles (professional and hierarchical), relationships, tools, time constraints and we add E-E climates. Questions guiding reflection might include: how are spaces and materials used to support shared decision-making? Is everyone heard? How are differences in power and privilege attended to within the organisation and team? How is data scrutinised by the team, using what criteria? How are emotions shared within the team? How does the meeting feel: rushed, relational?

### Epistemic cognition

Third, to the growing field of epistemic cognition, this study demonstrates the merits of conceptualising and studying epistemic cognition as not just individual, but social (Kelly, 2016) and, as we have shown, emotional practices. In much of the epistemic cognition research, experimental and quantitative methods are used to study cognitive processes and declarative knowledge reported via individual interviews or other self-reported methods (cf. Eastwood et al., 2017; Muis et al., 2015). Limited to no studies address how emotional climates interplay with the epistemic practices. Few if any studies focus on groups in naturalistic settings performing their everyday epistemic work. Our study design responds directly to calls for situated, naturally occurring research in the field of epistemic cognition (Kelly, 2016; Vogl et al., 2020). It also responds to specific calls to better understand epistemic cognition as a complex interactional phenomena in health professional education (Eastwood et al., 2017). Thus, analysing IPP and case conferences as situated epistemic cognition practices is apt and, to the best of our knowledge, novel.

Supporting this contribution, we offer insight into the methodological practices necessary to analysing social practices of epistemic cognition using naturalistic data. In this study, we developed a method of coding AIR transcript data by working backwards from the IPP achieved epistemic ends, to identify the processes they relied upon to generate those ends, what epistemic ideals were reflected in those processes, and what aims about knowledge and knowing these ideals implied. Although in some instances knowledge aims were clear from the outset of the IPP teams’ actions, it was more common to use our reverse coding method to identify the tacit nature of the AIR components.

Finally, in completing the analysis of our naturalistic data using Chinn et al.’s (2014) AIR model, it became evident that the epistemic practices used were often tacit and strongly reliant on the authority of the speaker (based in disciplinary expertise and/or recent in-depth engagement with the patient) (cf. Sandoval et al., 2016). Little overt effort was made to evaluate knowledge in a declarative way. Rather, the IPP team member’s memory, disciplinary authority, and recency of their contact with the patient were the dominant criteria tacitly applied to test veracity of knowledge contributions to the case conferences, working towards care plan development. This indicates that within teams of experts, the need to externalise criteria for evaluation and to set specific processes for producing knowledge may not be deemed necessary.

## Conclusion

Through this study, we advance a theory of IPP as involving *emotional-epistemic climates* (E-E climates) to explicate how knowledge is created, shared, and evaluated by clinicians in interprofessional case conferences, and how such practices are moderated by emotional dynamics. Our findings demonstrate that epistemic practices in IPP are based on a cumulation of experiences, interactions and expertise, embedded in memories and databases on laptops. What is more, we show that these practices are contingent on emotional climates, with more effervescent emotional climates prompting broader interprofessional participation. This theorisation supports a shift in epistemic cognition research, demonstrating the intersections in emotion and cognition as social and situated. It also supports calls to expand theorisation of IPP, with implications for IPE. Namely, our findings show the merits of foregrounding emotions in opening IPE curriculum to approaches underpinned by socio-materialist conceptualisations of IPP and learning, helping future health professionals to see and eventually co-create IPP as a relational practice situated within an E-E climate.

## Data Availability

The data that support the findings of this study are available from the authors, but restrictions apply to the availability of these data, which were used in alignment with ethics approval for the current study and so are not publicly available. The data are, however, available from the authors upon reasonable request and with the permission of the overseeing human research ethics committees.

## Footnotes

1 Said another way, judgements and deliberations about knowledge are social and emotional; this doesn’t mean that those doing the deliberating (in our study, clinicians in case conferences) treat information this way.

2 It is important to note that registrars are specialists in training whose presence in the department is only temporary, and thus their relationships with other team members are less established.
